# Supplementary material for: Mapping Geospatial Processes Affecting the Environmental Fate of Agricultural Pesticides in Africa
Source: Int J Environ Res Public Health. 2019 Sep 20;16(19):3523. doi: 10.3390/ijerph16193523 (PMC6801543; doi:10.3390/ijerph16193523)
Supplement: Supplementary file 1 [file ijerph-16-03523-s001.pdf]

Supplementary Materials

Table S1. In total, 24 pesticide fate models were selected. The variables that were used in each pesticide fate model are indicated by ‘x’ (Table 1).

	Model	Advection	Baseflow	By-pass flow	Convection	Deposition	Diffusion	Dispersion/ dissolution	Dissipation	Evapo- transpiration	Infiltration
1	BASINS										x
2	CASCADE-TOXSWA										x
3	Chemical fate model	x				x					x
4	CliMoChem	x					x				
5	CoZMo-POP-2 model	x						x		x	
6	CRACK-NP			x							x
7	Dynamic multimedia environmental fate model					x	x	x			x
8	EPIC									x	
9	GIBSI										x
10	GLEAMS								x	x	x
11	HSCTM-2D	x				x	x	x			
12	LEACHM									x	x
13	MACRO		x		x		x			x	x
14	OPUS									x	x
15	PEARL		x		x		x	x	x	x	x
16	PELMO		x					x			x
17	PESTLA		x		x			x			x
18	PLM			x			x			x	x
19	PRIMET									x	x
20	PRZM	x					x	x			x

21	RZWQM		x	x			x	x	x
22	SESOIL	x			x	x		x	x
23	SIMULAT			x			x	x	x
24	SWAT								x

	Model	Lateral throughflow	Percolation	Plant uptake	Sorption	Surface runoff	Transformation and degradation	Volatilization	Wash-off	Water erosion	Wind drift
1	BASINS		x		x	x			x		
2	CASCADE-TOXSWA		x			x				x	x
3	Chemical fate model		x			x	x		x		
4	CliMoChem						x	x			
5	CoZMo-POP-2 model	x		x	x	x	x	x	x		
6	CRACK-NP		x		x						
7	Dynamic multimedia environmental fate model		x				x	x			x
8	EPIC					x				x	x
9	GIBSI					x				x	
10	GLEAMS		x			x			x		
11	HSCTM-2D				x		x			x	
12	LEACHM		x	x	x	x					
13	MACRO	x	x		x	x	x				
14	OPUS		x			x	x			x	
15	PEARL		x	x			x	x	x		
16	PELMO		x	x	x	x	x	x	x	x	
17	PESTLA		x		x		x				
18	PLM		x		x		x				
19	PRIMET		x			x		x			x
20	PRZM		x	x	x	x	x			x	
21	RZWQM		x	x	x	x		x			

22	SESOIL	x	x	x	x	x	x	x
23	SIMULAT			x				
24	SWAT	x	x	x			x	x

Table S2. How often a variable was used in the selected pesticide fate models and the processes that were mapped in this study.

Variables	# models that use this variable	Processes that were mapped in this study
Infiltration	20	Leaching
Percolation	18	Leaching
Surface runoff	17	Surface runoff generation, transfer, accumulation
Transformation and degradation	13	
Sorption	13	Soil storage and filtering capacity
Evapotranspiration	12	Volatilization
Water erosion	9	Erosion
Volatilization	8	Volatilization
Diffusion	8	
Dispersion	7	
Wash-off	8	
Advection	6	
Plant uptake	6	
Base flow	5	
By-pass flow	4	
Deposition	4	
Dissipation	4	
Wind drift	4	
Convection	3	
Lateral through flow	2	

Table S3. Additional information on the existing geospatial datasets that were used in this study for creating maps of the processes associated with pesticide fate after spraying.

Property	Unit	Database	Spatial resolution	Spatial coverage	Temporal resolution	Temporal coverage	Source
Cation Exchange Capacity	cmol + /kg	SoilGrids	30-arc second	Sub-Saharan Africa	Static		[1]
Clay content	%	SoilGrids	30-arc second	Sub-Saharan Africa	Static		[1]
Depth to bedrock	cm	SoilGrids	30-arc second	Sub-Saharan Africa	Static		[1]
Elevation	m	SRTM-DEM	3 arc-second	Global	Static		[2]
Enhanced Vegetation Index	--	Weiss et al., 2014	30-arc second	Global	Static		[3]
Flow accumulation	m	HydroSHEDS	30 arc-second	Global	Static		[4]
Groundwater depth	m		30-arc second	Global	Static		[5]
Land use class	--	MCD12Q1	30-arc second	Global	Yearly	2001–2012	[6]
Potential Evapotranspiration	mm/month	CGIAR-CSI GeoPortal	30-arc second	Global	Static	Long-term average 1950-2000	[7]
Rainfall erosivity	MJ·mm/ha/ h/yr	USLE	30 arc-second	Global	Static		[8]
Relative humidity	%	Global Forecast System	15 arc-minute	Global	16-day	2015-present	[9]
Sand content	%	SoilGrids	30-arc second	Sub-Saharan Africa	Static		[1]
Silt content	%	SoilGrids	30-arc second	Sub-Saharan Africa	Static		[1]
Slope	°	SRTM-DEM	3 arc-second	Global	Static		[2]
Soil drainage class	--	AfSoilGrids250m	30-arc second	Sub-Saharan Africa	Static		[10]

Property	Unit	Database	Spatial resolution	Spatial coverage	Temporal resolution	Temporal coverage	Source
Soil moisture	mm	NASA-USDA SMAP Global Soil Moisture Data	15 arc-second	Global	3-days	2015 - present	[11]
Soil organic matter content	‰	SoilGrids	30-arc second	Sub-Saharan Africa			[1]
Soil pH in H_2O	--	SoilGrids	30-arc second	Sub-Saharan Africa	Static		[1]
Soil structure class	--	HWSD	30-arc second	Global	Static		[12]
Soil thickness	cm	S-World	30-arc second	Global	Static		[13]
Solar radiation	$kJ/m^2/day$	WorldClim V.2.	30-arc second	Global	Long-term monthly average	1950-2000	[14]
Stream length	m	HydroSHEDS	30 arc-second	Global	Static		[4]
Temperature	°C	MOD11A1 V6	30-arc second	Global	1-day	2000-present	[15]
Watershed area	m^2	HydroSHEDS	30 arc-second	Global	Static		[4]
Wind velocity	m/s	WorldClim V.2.	30-arc second	Global	Long-term monthly average	1950-2000	[14]

Table S4. An insecticide residue database was compiled from a literature review in Web of Knowledge. The table includes the search terms that were used to find studies that measured insecticide residues in soil, sediment, water and air. The following data were systematically extracted: year and month(s) of sampling, collection methods and depth, extraction method, quantification method, limit of quantification and detection, insecticide type and class, detected insecticide concentration and geographical coordinates.

‘pyrethroid’ AND spati*’ OR ‘pyrethroid’ AND ‘map*’
‘organophos AND spati*’ OR ‘organophos’ AND ‘map*’
‘carbamate’ AND spati*’ OR ‘carbamate’ AND ‘map*’
‘pyrethroid’ AND ‘watershed’ OR ‘run-off’ OR ‘groundwater’ OR ‘drift’ OR ‘deposition’ OR ‘precipitation’ OR ‘soil’ OR ‘sediment’ OR ‘coverage’ OR ‘atmosphere*’
‘organophos* AND ‘watershed’ OR ‘run-off’ OR ‘groundwater’ OR ‘drift’ OR ‘deposition’ OR ‘precipitation’ OR ‘soil’ OR ‘sediment’ OR ‘coverage’ OR ‘atmosphere*’
‘carbamate AND ‘watershed’ OR ‘run-off’ OR ‘groundwater’ OR ‘drift’ OR ‘deposition’ OR ‘precipitation’ OR ‘soil’ OR ‘sediment’ OR ‘coverage’ OR ‘atmosphere*’
‘residu*’ AND ‘pyrethroid’ OR ‘organophos*’ OR ‘carbamate’ AND ‘COUNTRYNAME’ NOT ‘indoor residual spray*’

References

1. Hengl T, Mendes de Jesus J, Heuvelink GBM, Ruiperez Gonzalez M, Kilibarda M, Blagotic A, et al. SoilGrids250m: Global gridded soil information based on machine learning. PLoS One. 2017;12:e0169748.

2. Jarvis A, Reuter HI, Nelson A, Guevara E. Hole-filled SRTM for the globe Version 4. CGIAR-CSI SRTM 90m Database. 2008. Available from: http://srtm.csi.cgiar.org.

3. Weiss DJ, Atkinson PM, Bhatt S, Mappin B, Hay SI, Gething PW. An effective approach for gap-filling continental scale remotely sensed time-series. ISPRS J Photogramm Remote Sens. 2014;98:106–18.

4. Lehner B, Verdin K, Jarvis A. New global hydrography derived from spaceborne elevation data. Eos, Trans Am Geophys Union. 2008;89:93–4.

5. Fan Y, Li H, Miguez-Macho G. Global patterns of groundwater table depth. Science 2013; 339: 940-943.

6. Channan S, Collins K, Emanuel WR. Global mosaics of the standard MODIS land cover type data. University of Maryland and the Pacific Northwest National Laboratory, USA. 2014. Available from: http://glcf.umd.edu/data/lc/.

7. Trabucco A, Zomer RJ. Global Aridity Index (Global-Aridity) and Global Potential Evapo-Transpiration (Global-PET) Geospatial Database. CGIAR-CSI GeoPortal. 2009. Available from: https://cgiarcsi.community/data/global-aridity-and-pet-database/.

8. P Panagos P, Borrelli P, Meusburger K, Yu B, Klik A, Jae Lim K, et al. Global rainfall erosivity assessment based on high-temporal resolution rainfall records. Sci Rep. 2017;7:4175.

9. Kleist DT. An evaluation of hybrid variational-ensemble data assimilation for the NCEP GFS. University of Maryland-College Park; 2012.

10. Hengl T, Heuvelink GBM, Kempen B, Leenaars JGB, Walsh MG, Shepherd KD, et al. Mapping Soil Properties of Africa at 250 m Resolution: Random Forests Significantly Improve Current Predictions. PLoS One. 2015; 25;10:e0125814.

11. Mladenova IE, Bolten JD, Crow WT, Anderson MC, Hain CR, Johnson DM, et al. Intercomparison of Soil Moisture, Evaporative Stress, and Vegetation Indices for Estimating Corn and Soybean Yields Over the U.S. IEEE J Sel Top Appl Earth Obs Remote Sens. 2017;10:1328–43.

12. Fischer G, Nachtergaele F, Prieler S, Van Velthuizen, H.T. Verelst L, Wiberg D. Global Agro-ecological Zones Assessment for Agriculture (GAEZ 2008). IIASA, FAO. 2008. Available from: http://www.fao.org/soils-portal/soil-survey/soil-maps-and-databases/harmonized-world-soil-database-

v12/en/.

13. Stoorvogel JJ, Bakkenes M, Temme AJAM, Batjes NH, ten Brink BJE. S-World: A Global Soil Map for Environmental Modelling. L Degrad Dev. 2017;28:22–33.

14. Fick SE, Hijmans RJ. WorldClim 2: new 1-km spatial resolution climate surfaces for global land areas. Int J Climatol. 2017;37:4302–15.

15. Wan Z, Hook S, Hulley G. MOD11A1 MODIS/Terra Land Surface Temperature/Emissivity Daily L3 Global 1km SIN Grid V006. NASA EOSDIS LP DAAC. 2015. Available from: https://lpdaac.usgs.gov/node/819.
